# Role of Matrix-Associated Autologous Chondrocyte Implantation with Spheroids in the Treatment of Large Chondral Defects in the Knee: A Systematic Review

**DOI:** 10.3390/ijms22137149

**Published:** 2021-07-01

**Authors:** Lucienne Angela Vonk, Giulietta Roël, Jacques Hernigou, Christian Kaps, Philippe Hernigou

**Affiliations:** 1 CO.DON AG, 14513 Teltow, Germany; l.vonk@codon.de (L.A.V.); g.roel@codon.de (G.R.); c.kaps@codon.de (C.K.); 2 Department of Orthopaedics, University Medical Center Utrecht, Utrecht University, 3584 CX Utrecht, The Netherlands; 3 Department of Orthopedic Surgery, EpiCURA Hospital, 7331 Baudour, Belgium; Jacques.hernigou@gmail.com; 4 Laboratory of Bone and Metabolic Biochemistry, Faculty of Medecine, Université Libre de Bruxelles, 1070 Brussels, Belgium; 5 Department of Orthopaedic Surgery, Faculty of Medicine, UPEC (University Paris-Est, Créteil), 94000 Créteil, France

**Keywords:** autologous chondrocyte implantation, spheroids, Spherox, cartilage defects, advanced therapy medicinal product, preclinical research

## Abstract

Autologous chondrocyte implantation (ACI) is a cell therapy for the treatment of focal cartilage defects. The ACI product that is currently approved for use in the European Union (EU) consists of spheroids of autologous matrix-associated chondrocytes. These spheroids are spherical aggregates of ex vivo expanded human autologous chondrocytes and their self-synthesized extracellular matrix. The aim is to provide an overview of the preclinical and nonclinical studies that have been performed to ensure reproducible quality, safety, and efficacy of the cell therapy, and to evaluate the clinical data on ACI with spheroids. A systematic review was performed to include all English publications on self-aggregated spheroids of chondrocytes cultured in autologous serum without other supplements. A total of 20 publications were included, 7 pre- and nonclinical and 13 clinical research publications. The pre- and nonclinical research publications describe the development from concept to in vivo efficacy and quality- and safety-related aspects such as biodistribution, tumorigenicity, genetic stability, and potency. The evaluation of clinical research shows short- to mid-term safety and efficacy for the ACI with spheroid-based treatment of cartilage defects in both randomized clinical trials with selected patients, as well as in routine treatment providing real-world data in more complex patients.

## 1. Introduction

Articular cartilage is the cartilage that covers the end of bones in synovial joints. It provides a smooth surface for the movement of articulating bones, and it allows to withstand compressive and shear forces. This cartilage is hyaline cartilage that contains only a small number of cells, chondrocytes (<10%), and the extracellular matrix is composed of collagens, mainly type II collagen, and glycosaminoglycan containing proteoglycans. It has no blood supply and is not innervated by nerves or lymphatic vessels [1].

Focal cartilage defects can cause symptoms such as pain, swelling, stiffness, giving way, or locking of the knee affecting quality of life. As cartilage has a very limited ability to repair itself, these defects require surgical treatment. Moreover, if cartilage defects are left untreated, the damage progresses leading to osteoarthritis [2]. Osteoarthritis is a whole-joint disease characterized by progressive cartilage and meniscus degradation, synovitis, the formation of osteophytes, and thickening of the subchondral bone [3,4].

Autologous chondrocyte implantation (ACI) is a cell therapy that is used for the treatment of medium to larger sized (>2 cm^2^) focal cartilage defects. ACI has been used for over 30 years and it provides good to satisfactory results and it is a well proven treatment with level 1 evidence [5,6]. ACI is a two-step procedure. In a first surgery, a biopsy of healthy cartilage from a non-weight bearing site of the cartilage is taken. Subsequently, chondrocytes are isolated from the biopsy and culture expanded. In a second surgery, the culture-expanded autologous chondrocytes are implanted in the cartilage defect. By implanting chondrocytes directly into the defect, the defect will be filled with new hyaline cartilage tissue.

**Figure 1:**
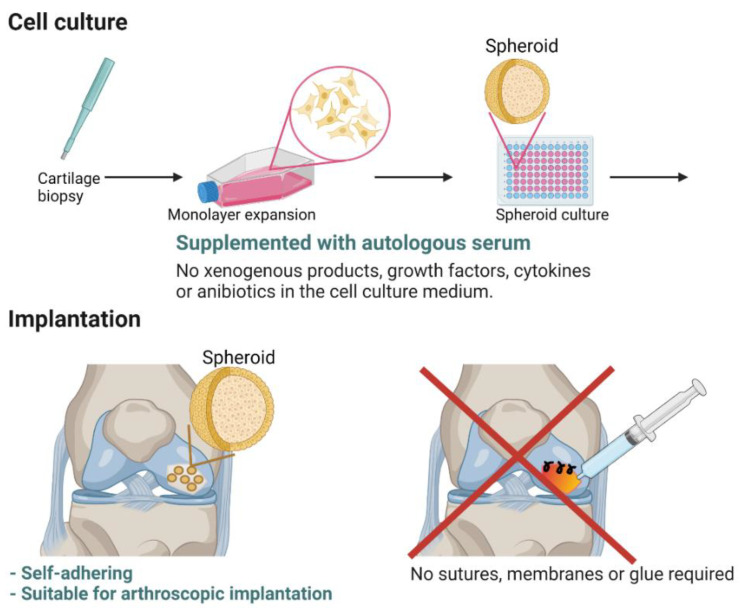
For the manufacturing of ACI with spheroids, autologous chondrocytes are culture expanded and subsequently cultured as spheroids using culture medium supplemented with only autologous serum and without other supplements such as growth factors, cytokines, and or antibiotics. The spheroids are self-adhering to subchondral bone, allowing for arthroscopic implantation and omitting the need for sutures, membranes, and glue. This figure was created with BioRender.com.

Regulatory wise, ACI is an advanced therapy medicinal product (ATMP). Thus, extensive preclinical, nonclinical, and clinical studies need to be performed to ensure reproducible quality, safety, and efficacy of the cell therapy. Currently, Spherox (CO.DON AG, Teltow, Germany) is approved for the repair of symptomatic articular cartilage defects of the femoral condyle and the patella of the knee (International Cartilage Repair Society (ICRS) grade III or IV) with defect sizes up to 10 cm^2^ in adults in the EU. Spherox has been used under the name chondrosphere since 2004 in Germany and changed to Spherox after obtaining the EU-wide marketing authorization. Chondrosphere is currently still used in Germany for joints other than the knee. Spherox contains spheroids of autologous matrix-associated chondrocytes. The spheroids are spherical aggregates of ex vivo expanded human autologous chondrocytes and their self-synthesized extracellular matrix. The novelty of this ACI with spheroids is that both the cell culture and the implantation of the ACI are fully autologous (Figure 1). The cell cultures are performed with autologous serum without other supplements such as growth factors, cytokines, and/or antibiotics. In addition, the spheroids are self-adhesive to the subchondral bone and therefore, the implantation does not require any sutures, membranes, covers, or glue [7]. 

The purpose of this review is to systematically evaluate the available literature on fully autologous ACI with spheroids. As such, an overview of the available preclinical and nonclinical data is provided, covering required quality aspects related to the manufacturing process. Furthermore, the clinical data on the use of ACI with spheroids for the treatment of cartilage defects in the knee are documented and analyzed.

## 2. Methods

A review of the literature was performed according to the Preferred Reporting Items for Reviews and Meta-analyses (PRISMA) guidelines. A search was conducted on 30 April 2021, in the electronic databases of PubMed and Web of Science, using the terms “autologous chondrocyte implantation AND spheroid”, “ACI AND spheroid”, “Spherox”, “chondrosphere”, “autologous chondrocyte implantation AND CODIS”, “autologous chondrocyte implantation AND COWISI”, and “cartilage defect regeneration AND microtissue”. CODIS and COWISI were the coding names of the phase II and III clinical trials, respectively, standing for CO.DON dose investigation study (CODIS) and CO.DON wirksamkeit und sicherheit, which translates into effectiveness and safety (COWISI). 

The criteria were original research publications (excluding conference abstracts and publications), written in English, that described either clinical studies with matrix-associated autologous chondrocyte implantation with spheroids in the knee or nonclinical research on self-aggregated spheroids (by the use of agarose coated wells) of chondrocytes cultured in autologous (or surrogate autologous) serum without other supplements such as growth factors, cytokines, and antibiotics. These criteria were chosen as the self-aggregation of chondrocytes into spheroids and the completely autologous cell culture and implantation (Figure 1) are main features of ACI with spheroids. 

Citations were examined for their relevance and to determine their eligibility for inclusion. 

This review was performed according to the Preferred Reporting Items for Systematic Reviews and Meta-Analyses (PRISMA) guidelines, which provides an evidence-based minimum set of items for reporting in systematic reviews and meta-analyses. One of these items is to assess the risk of bias of included publications, or in other words to describe the likelihood that features of the design or conduct of the study will give misleading results. For randomized controlled trials (RCTs), the modified Cochrane Risk of Bias Tool is a well-known and appropriate risk of bias assessment tool and this was used to assess the included RCTs individually for risk of bias (Table 1) [8]. As the design of nonrandomized studies is different compared to RCTs, other tools to assess the risk of bias can be more appropriate. Therefore, the nonrandomized studies were evaluated via the (MINORS) criteria (Table 2) [9].

Levels of evidence: 1a: Systematic reviews (with homogeneity) of randomized controlled trials; 1b: Individual randomized controlled trials (with narrow confidence interval); 1c: All or none randomized controlled trials; 2a: Systematic reviews (with homogeneity) of cohort studies; 2b: Individual cohort study or low quality randomized controlled trials (e.g., <80% follow-up); 2c: “Outcomes” Research; ecological studies; 3a: Systematic review (with homogeneity) of case-control studies; 3b: Individual case-control study; 4: Case-series (and poor-quality cohort and case-control studies); 5: Expert opinion without explicit critical appraisal, or based on physiology, bench research or “first principles”.

## 3. Results

**Figure 2:**
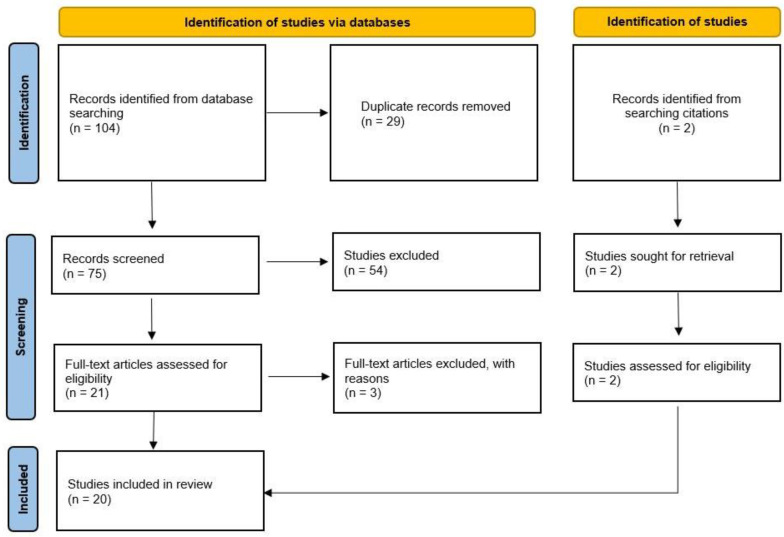
Preferred reporting items for systematic reviews and meta-analyses (PRISMA) flow diagram: Summary of literature search.

The literature search yielded 34 publications in PubMed and 70 in Web of Science. After removing 29 duplicates and screening the titles and abstracts, the full texts of 21 publications were screened, after which 18 [7,10,11,12,13,14,15,17,18,19,20,21,22,23,24,25,26,27] were included and 3 [28,29,30] were excluded due to the use of xenogeneic materials [28] or antibiotics [29] in the cell culture, or the spheroids were formed by pelleting cells with centrifugation [30]. Two additional articles [16,31] were identified from citations (Figure 2). Thus, a total of 20 publications were included. Five of these publications describe two randomized controlled trials: A dose confirmation trial and a comparative trial against microfracture (MFx). In general, the risk of bias was low in those trials, except for the blinding. In the phase II trial, the patients were blinded to the dose level, but the treating physician not as the dose of implanted spheroids needed to be verified. The MRI images were analyzed by an independent radiologist without knowledge of the dose and time between treatment and MRI. In the phase III trial, the patients and the physician could not be blinded due to the nature of the treatments (one surgery for MFx versus two for ACI). However, the assessment of the MRI images and histology were performed by an independent radiologist and pathologist, who were blinded for the treatment. The nonrandomized studies included prospective and retrospective cohort studies, retrospective case series, and subgroup analyses from the randomized studies. 

### 3.1. Preclinical and Nonclinical Studies—From Concept to Advanced Therapy Medicinal Product

**Figure 3:**
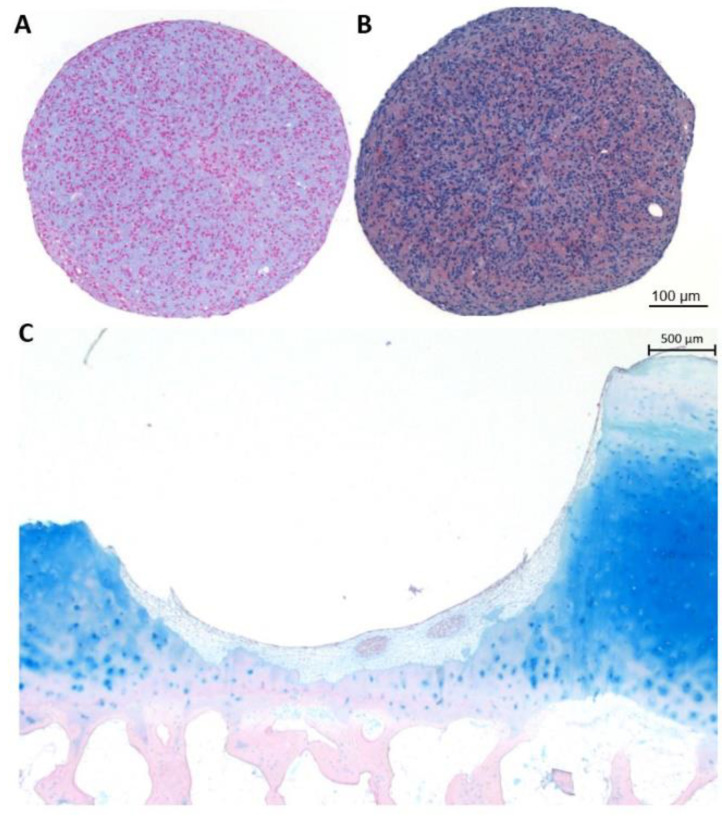
Alcian blue staining (**A**) and aggrecan immunostaining (**B**) on sections of 10 day-cultured spheroids. Alcian blue staining on sections of an in vitro co-culture of spheroids in a chip of human osteochondral tissue (**C**). This is an original figure and not a reproduction.

The concept of spheroids derived from self-aggregated chondrocytes was first presented by Anderer and Libera in 2002 [7]. They showed that it was feasible to create spheroids by culturing expanded chondrocytes on agarose-coated wells (Figure 3). The complete cell culture was performed in culture medium supplemented with human serum and without the use of additional growth factors, antibiotics, or other xenogeneic products. Moreover, the spheroids were self-adhesive to cartilage and subchondral bone, the chondrocytes could migrate from the spheroids, spheroids could fuse, and chondrocytes in the spheroids were able to deposit hyaline cartilage components. 

In a subsequent study, as a first proof-of-principle experiment, spheroids were implanted in a cartilage defect that was created in explants of macroscopically healthy osteochondral tissues obtained from patients undergoing total knee arthroplasty (Figure 3) [25]. These constructs were implanted subcutaneously in severe combined immunodeficiency (SCID) mice. After 4, 12, and 24 weeks, the implants were retrieved and subjected to histological analyses. There was an increase over time in the deposition of proteoglycans. Expression of the hyaline cartilage marker S100 was observed and stable at each time point. Type II collagen was deposited at 4 weeks and more intense staining was found at 12 weeks and at 24 weeks the intensity was similar to the surrounding cartilage. Type I collagen was also observed at all timepoints, but slightly decreased at 12 weeks. However, while type I collagen was more observed in certain locations, type II collagen was homogeneously present in the repair tissue and stronger than the type I collagen staining. Besides, integration of the repair tissue, defined as the percentage of the chondrosphere surface adhering to the cartilage lesion borders, was assessed. Due to geometrical reasons, integration could not reach 100%. Integration increased from approximately 37% to 46% and 61% at 4, 12, and 24 weeks approximately. The morphology of the chondrocytes in the spheroids was still mixed at 4 weeks with chondrocytes showing a typical round chondrocytic morphology and more elongated fibroblast-like cells. At 12 and 24 weeks, almost all cells presented with a typical chondrocytic morphology. This study demonstrated, for the first time, the intended function of the chondrocyte spheroids to fill up a cartilage lesion.

The in vivo efficacy of chondrocyte spheroids in a large animal model was evaluated in a study on Göttinger minipigs under Good Manufacturing Practice (GMP) [23]. Cartilage was obtained from the upper talocrural joint of five skeletally mature male Göttinger pigs for chondrocyte expansion and spheroid formation in culture medium supplemented with 10% autologous serum. After about eight weeks, two defects were created in the trochlear grooves of each animal and 20–30 spheroids were implanted in the experimental defects using 20 min self-adherence to the subchondral bone. The defects of two animals were side matched and left untreated as control. One week after treatment, the animals moved normally. After two months, the treated and control defects were prepared for (immuno)histological assessment. During explantation, the joints appeared normal and without signs of synovitis. Some self-repair was observed in the untreated defects, but the defects treated with spheroids demonstrated a higher degree of defect filling accompanied by a smoother regenerated surface. The control defects were filled with a combination of fibrocartilage and hyaline cartilage; the investigators observed no deposition of proteoglycans and a moderate expression of type I collagen, and one control defect showed a moderate expression of type II collagen. The repair tissue in the spheroid treated defects was hyaline cartilage with deposition of proteoglycans and type II collagen, while hardly any type I collagen was observed. 

The safety issue of chondrocyte spheroids after implantation was addressed by evaluating biodistribution and tumorigenicity evaluated in NSG mice under Good Laboratory Practice (GLP) [26]. Spheroids, which were additionally produced from chondrocytes of female patients receiving treatment with Chondrosphere, were subcutaneously implanted in NSG mice. Spheroids produced from the mouse embryonic fibroblast cell line NIH/3T3 and the human colorectal adenocarcinoma cell line Caco-2 were used as references in the tumorigenicity study. After 31 ± 1 days, mice were euthanized for the biodistribution study. In 39/40 mice, the spheroids were identified by eye and in 5/39 mice, the spheroids were fragmented. Using immunohistochemistry (IHC) for HLA-ABC 4 mm discs containing the spheroids, no signs of active cell migration were observed, but 17 samples were excluded from IHC evaluation due to technical uncertainties. In addition, qPCR for human DNA was performed on 10 mm discs. Seven of these discs were positive for human DNA, although two for male DNA. However, for these seven samples, the 20 mm discs were also evaluated by PCR, which were all negative for human DNA. From the 17 samples that were excluded from IHC evaluation, seven animals were randomly chosen to perform additional qPCR on organs. These were all negative, so there was no suspicion of biodistribution of spheroid-derived chondrocytes. 

For the tumorigenicity study, all mice implanted with chondrocyte spheroids had good health scores, no weight loss, and were euthanized after 180 ± 5 days post implantation according to the initial planning. All mice implanted with Caco-2 spheroids had to be euthanized 22- or 23-days post-implantation due to bad health scores and they all had developed tumors at the implantation site. Additionally, all animals implanted with NIH/3T3 also developed tumors and 6/8 mice had to be euthanized between day 84 and 167 due to bad health scores. From the chondrocyte-spheroid implanted mice, 5/40 mice showed abnormalities upon organ and tissue dissection. Two abnormalities were hair retention cysts, one was a microfocal benign malformation, and two were tumors that were likely spontaneous murine tumors as they were HLA-ABC negative. Thus, there was no indication of increased tumor frequency upon use of chondrocyte spheroids. 

In order to bring the chondrocyte spheroids as a medicinal product to the market, product specifications were defined, and release criteria were justified as part of the quality control strategy according to the ICH guideline Q6B. Critical Quality Attributes (cQAs) for identity, impurity and potency needs to be defined and tests established based on the cQAs to ensure consistent quality and to ensure the intended function of the final product.

To develop a potency release test for Spherox, spheroids were produced from 14 chondrocyte donors [24]. For each donor, basic spheroid characteristics such as proteoglycan production and release into the culture medium, protein expression of aggrecan, and gene expression of cartilage acidic protein 1 (CTRAC1), S100B, and aggrecan (ACAN) were determined. Besides, the spheroids were implanted in a cartilage defect that was created in explants of macroscopically healthy osteochondral tissues obtained from patients undergoing total knee arthroplasty (Figure 3). These constructs were cultured in vitro for 12 weeks. The regeneration capacity, measured by the amount of formed repair tissue, that did contain hyaline cartilage components, was determined, and correlated to the basic characteristics of the spheroids. A positive correlation between the regenerative capacity and aggrecan protein and mRNA expression in the spheroids before implantation was observed, demonstrating that aggrecan protein expression levels in spheroids before implantation can predict the biological activity of the product, and therefore can potentially be used as a release test for potency. 

To establish a robust and consistent manufacturing process that delivers products of high-quality, critical process parameters have been identified that could have a negative effect on the quality of the final product. Therefore, since extensive in vitro expansion can cause genetic instability and chromosomal aberrations [32], the genetic stability of the chondrocytes was evaluated [27]. Chondrocytes were expanded in a monolayer up to passage 10, and at passages 2, 3, 4, 5, and 10, spheroids were produced. The genetic stability of both the monolayers and the spheroids was evaluated with GTG banding (karyotyping) and SKY (spectral karyotyping). Well within the expansion limits used for Spherox, the monolayers and spheroids did not show any clonal genetic instability until passage 3. More extensive expansion could cause genetic aberrations that are inherited during the next cell duplication [27]. 

Finally, the effects of the cell cultivation times on clinical outcome were assessed [31]. It is known that chondrocytes dedifferentiate during expansion in monolayer. This can affect the regenerative capacity of expanded chondrocytes and thus also clinical outcome [33]. It was observed that longer cultivation time of passage 0 of the spheroids and the total cultivation time (monolayer expansion and spheroid culture) negatively correlate with the primary clinical outcome score of patients included in the phase II and phase III clinical trials. In addition, a subgroup with shorter total cultivation time contained less non-responders than a subgroup with longer total cultivation time. Thus, implementing more stringent cell culture criteria can improve the clinical outcome of ACI with spheroids [31]. 

### 3.2. Clinical Studies

An overview of all the clinical studies and their outcomes is presented in Table 3. The overall clinical outcomes of patients who underwent ACI with spheroids showed scores indicative of significant and clinically relevant improvement (Table 4). In addition, magnetic resonance imaging (MRI) analyses and magnetic resonance observation of cartilage repair tissue (MOCART) scores showed mostly complete defect fill with good integration into the surrounding tissue and repair with hyaline cartilage-like tissue (Table 4). These findings were supported by second-look arthroscopies and macroscopic cartilage repair assessment (Table 3). Moreover, a limited number of biopsies were obtained from the repair tissue of a variety of patients and most of the repair tissue had hyaline cartilage characteristics as determined by histology and immunohistochemistry (Table 3).

The phase II clinical trial was a prospective, single-blinded, multicenter, dose-confirmation study in which the safety and efficacy of three different doses of Spherox for the treatment of cartilage defects in the knee were investigated (ClinicalTrials.gov identifier: NCT01225575, EudraCt No. 2009-016816-20) [10,11,12]. The primary outcome was the increase in the overall Knee Injury and Osteoarthritis Outcome Score (KOOS) at 12 months follow-up, which was improved from mean 57.0 ± 15.2 to 73.4 ± 17.3 (Table 3) [10]. An improvement was also found for all KOOS subscales, international knee documentation committee (IKDC), and MOCART score at 12 months follow-up, which was stable over the follow-up time (Table 3) [10]. Furthermore, no differences could be found between the dose groups. At four years follow-up, a treatment failure rate of 4% was found (three out of 75 patients) [12]. The most reported treatment-related adverse events were joint effusion, arthralgia, and swelling. No relation could be observed between the frequency, onset, duration, or severity of adverse reactions and the dose [11,12].

The phase III clinical trial was a prospective multicenter study to compare the efficacy and safety of treating cartilage defects with ACI using Spherox to treatment with MFx (ClinicalTrials.gov identifier: NCT01222559, EudraCt No. 2009-016466-82) [13,14]. Primary outcome was the change in overall KOOS at 24 months follow-up compared to baseline. The overall KOOS improved from 56.5 ± 15.4 at baseline to 81.5 ± 17.3 at 24 months follow-up in the ACI group and from 51.7 ± 16.5 at baseline to 73.2 ± 18.8 at 24 months in the MFx group (Table 3) [13]. An improvement was also found for all KOOS subscales and MOCART score at 24 months follow-up, which was stable over the follow-up time (Table 3) [14]. Non-inferiority testing showed that ACI with spheroids was non-inferior to MFx and KOOS activities of daily living (ADL) showed superiority of ACI over MFx at the descriptive level at 24 months [13], and KOOS ADL and KOOS sports and recreation (Sport/Rec) at 36 months [14]. Considering safety, as in the phase II study, the most frequently reported adverse reactions were joint effusion, arthralgia, and joint swelling, for both the ACI and the MFx group. No likely association with either treatment was revealed and no (new) safety concerns were found [14]. 

Two publications focused on the treatment of patients with cartilage defects on the patella (which was a subgroup analysis of the phase II clinical trial) [21] and/or defects due to recurrent patellar dislocation in combination with medial patellofemoral ligament reconstruction [16]. Both studies showed that the treatments of the patella defects were successful and the increase of overall KOOS for patella patients was non-inferior to patients with a defect on the femur. 

Moreover, many patients have been treated with ACI with spheroids as routine treatments and thus also in combination with concomitant procedures, either at biopsy or at implantation. These concomitant surgeries consisted mainly of high tibial osteotomy or medial tibial tubercle transfer, anterior cruciate ligament reconstruction, and meniscal surgery. In a study with adolescents, bone grafting was one of the main concomitant surgeries as approximately 50% of these patients had an osteochondral defect due to osteochondritis dissecans [19]. It has to be noted here that the adolescent patients were treated with chondrosphere in Germany under the German regulations. ACI with spheroids provided good patient-reported outcome scores [15,16,17,18,19], MRI [15,16,18,19], macroscopic [17,22], and histological [20] evaluations in patients with a routine treatment (Table 3). 

## 4. Discussion

The clinical data reported on ACI with spheroids demonstrated promising short- to mid-term results with meaningful improvement and good-quality repair, even in more challenging patients. The preclinical and nonclinical studies reported the concept and in vivo safety and efficacy, accompanied by a partial overview of requirements to produce a safe cell based ATMP with reproducible quality. 

### 4.1. Quality in Manufacturing

Manufacturing an autologous, cell based ATMP that meet the international standards of the (European) regulatory authorities is challenged by various factors. Because of the autologous nature of the starting material, the manufacturing process is subject to variability of the biopsy quality and as a direct result, the operational ranges of process parameter as well as a wide range of the test results regarding quality control. These factors could potentially influence the quality of the cells and/or the final product and hence the clinical outcome after ACI treatment [33]. In the case of an ACI, critical process parameters include the duration of biopsy digestion, cell yield obtained from the biopsy, cell expansion and cell cultivation time. Critical quality attributes are a prerequisite from the regulatory guidelines for ATMPs to characterize the product and are the basis of a decisive quality control strategy. They include parameters for safety, identity, impurity, and potency. 

Safety studies in mice addressing biodistribution and tumorigenicity indicated that ACI with spheroids is safe [26]. Although there is no indication available in the literature that cells would cause tumorigenesis after being expanded ex vivo for a limited increase of the number of cells [34], it is known that cell expansion can cause numerical and structural chromosomal aberrations [32]. Therefore, it is recommended to include safety studies regarding genetic stability to minimize risks of generating aberrant cells for ACI. An additional nonclinical study could confirm that expanding chondrocytes well within the range of Spherox yielded genetically stable cells [27]. 

Considering potency, it was shown that aggrecan protein and mRNA expression in spheroids before implantation correlated to the regenerative capacity of spheroids implanted in an in vitro cartilage defects model that can be used as a functional assay. Therefore, assessment of aggrecan mRNA expression levels in spheroids before implantation by means of quantitative PCR can be used as surrogate potency assay [24]. This conversion of the potency assay into a surrogate potency assay that will serve as a fast release test is essential due to time constraints. Without any preservation step in the manufacturing process, between the release test and the actual release of the final product, only 14 days are (or: limited time is) available for testing, including assessment of the test results. With respect to Matrix-induced Autologous Chondrocyte Implantation (MACI (Sanofi/Genzyme, withdrawn from use in the EU, currently approved for use in the US (Vericel)), it was reported that aggrecan gene expression could be used as a potency marker [35]. MACI cultured chondrocytes expressed relatively high levels of the aggrecan gene compared to dermal fibroblasts, and when these chondrocytes were cultured in 3D (pellets or alginate), the chondrocytes produced type II collagen. For ChondroCelect (TiGenix, withdrawn from use in the EU) it was reported that a set of molecular markers correlated with the cartilage-forming capacities of chondrocytes in an in vivo Ectopic Cartilage Forming Assay [36,37]. 

### 4.2. Clinical Use in the Knee

ACI with spheroids distinguishes itself from other ACIs (such as [38,39,40]) as it can be a fully autologous cell product. In the production of the cells, a medium supplemented with autologous serum is used and the cells are not exposed to any other growth factors, cytokines, and antibiotics [7]. This increases safety, for instance cell culture in medium supplemented with fetal bovine serum can lead to xeno-immunization and transmission of bovine pathogens, and pooled platelet lysate is associated with the same risks as blood transfusion such as an allergic reaction. As the spheroids are self-adhesive to the subchondral bone by extracellular matrix interactions, there is no need for additional cell carriers, covers, membranes, or sutures [15]. Therefore, the spheroids can be implanted with minimal invasiveness with an arthroscopy [22]. In addition, xenogenous scaffold material can interact with human cells and affect their performance [41]. Moreover, arthroscopic implantation of scaffolds and membranes can be complicated, and cells can undergo many stresses leading to cell death [42]. In addition, fixation by sutures does permanently injure the surrounding cartilage [43] and using human fibrin sealant to glue scaffolds can invoke immunological reactions, mainly due to the aprotinin [44]. The omission of xenogenous substances and materials during cell culture and implantation also makes ACI with spheroids suitable for patients who do not want to receive xenogenous products, for instance due to religion or lifestyle. 

Although autologous chondrocyte implantations have been used for over 30 years, the optimum dose has never been determined. The recommendation of using a concentration between 1 and 3 million cells per cm^2^ defect was based on satisfactory outcomes from the first case series of suspension-based ACI [5]. During the natural development of cartilage, mesenchymal precursor cells condensate and differentiate into chondrocytes, which then rapidly proliferate and produce cartilage specific extracellular matrix components [45]. Subsequently, the proliferation halts and part of the chondrocytes die by apoptosis, leading to a relative decrease in cell density cartilage tissue. This suggests that higher initial cell densities might improve neo-cartilage formation, but contradictory results have been found in in vitro studies [46,47,48]. Therefore, there was a clear need to investigate the effects of cell density for ACI in a clinical study. Three different spheroid doses were evaluated in the phase II study: A low dose with 3–7 spheroids/cm^2^, a medium dose of 10–30 spheroids/cm^2^, and a high dose with 40–70 spheroids/cm^2^ defect. However, no differences could be observed between the various dose groups [10,11,12]. Thus, the minimum required dose and the dose leading to an overdose are still unknown. 

The repair of symptomatic articular cartilage defects of the patella is included in the indication of Spherox. About one-third of all cartilage defects is located on the patella [49]. However, they are complex to treat due to the accessibility and biomechanics of the patella femoral joint [50]. In addition, lower clinical outcomes, lower success rate, and shorter durability have been reported for ACI to treat patella defects compared to defects in the femoral condyle [40,51]. On the other hand, some studies showed very promising results regarding patella cartilage defect repair [52,53]. As the phase II clinical study included relatively many patients with patellar defects, a subgroup analysis was performed to compare treatment of patella defects (45 patients) with the treatment of femur defects (28 patients) [21]. Overall, the patients and defect characteristics and cell dose were comparable between the patella and femur subgroups. The overall safety assessment suggested similar profiles between the patella and femoral subgroups up to 60 months follow-up. The overall KOOS and all KOOS subscores showed an improvement at 12 months follow-up, which was stable over 60 months. In addition, a non-inferiority analysis was performed for the overall KOOS at 12 months showing that the increase in overall KOOS for the patella subgroup was non-inferior to the increase in the femur subgroup. These analyses were performed on patients included in a randomized clinical trial and these patients had generally stable and well-aligned knees. Moreover, data from routine treatments also showed that ACI with spheroids is promising in the treatment of patella defects [16,18,19]. However, underlying pathologies that cause patella defects or affect their repair do need to be treated as well. In this way, good clinical results have been obtained of ACI with spheroids for patella defects in combination with tibial tubercle osteotomy and medial patellofemoral ligament repair and reconstruction [16].

As trauma is the major cause of cartilage defects in adults, cartilage defects often occur in combination with other joint trauma and many concomitant procedures are performed with ACI. With respect to this, the non-randomized studies showed that ACI with spheroids can be successful in combination with, besides tibial tubercle osteotomy and medial patellofemoral ligament repair and reconstruction, meniscus and anterior cruciate ligament repair, lateral release, and high tibial osteotomy [15,17,18,19]. In addition, the combination of bone grafting and ACI with spheroids was also successful in adolescents with osteochondritis dissecans [19]. The bone grafting was performed during the biopsy taking and with the implantation, without problems on the adherence of the spheroids. Thus, this shows promise that ACI with spheroids can be used for osteochondritis dissecans and other deep osteochondral lesions. 

### 4.3. Usage in Hip and Shoulder

The option of arthroscopic implantation makes ACI with spheroids very suitable for use at locations or other joints with difficult surgical access, such as the hip. Four case series were published on the repair of cartilage defects in the hip using ACI with spheroids [54,55,56,57]. Most of these cartilage defects in the hip were caused by cam-type femoroacetabular impingement. All studies reported on good clinical outcomes and improvement. In a few patients, a second-look arthroscopy was performed that showed complete defect fill with good integration of the repair tissue with a hyaline cartilage macroscopic appearance [56]. In addition, no differences were observed in a comparison with a hydrogel-based ACI (Novocart Inject (TETEC)) [55]. Besides the hip, one case series was published on the use of ACI with spheroids for the treatment of cartilage defects of the humeral head [58]. It was shown that the treatment provided satisfactory clinical results with a short- to mid-term follow-up time. In addition, as shown by MRI and second-look arthroscopy for a selection of patients, there was almost complete defect fill and only minor radiologic degenerative changes. So, although Spherox is only approved for the use in cartilage defects in the knee, the treatment of ACI with spheroids has shown good and promising results in other joints as well. 

## 5. Conclusions

In this review, several pre- and nonclinical studies have been performed on the concept of these spheroids: They have demonstrated in vivo safety and efficacy, and quality aspects for the safety and efficacy of the ACI product related to the manufacturing process. The clinical studies demonstrated cartilage regeneration and showed good results of the treatment of cartilage defects with the fully autologous and minimally invasive ACI with spheroids. The outcome parameters of the clinical studies included various patient reported outcome measures, MRI analyses, macroscopic repair assessments during second look arthroscopies, and histological analyses on biopsies taken from the repair tissue. 

With few but positive studies available for ACI products in the different joints, clinical benefits for the patient include decrease donor site morbidity when compared to mosaicplasty; it is also the only technique available for large defects in population or countries where allograft is unavailable. Given this, we can recommend ACI products for large (up to 10 cm^2^) chondral defects without important subchondral change. Furthermore, for those patients who have failed a previous repair with another surgical technique, this technique with autologous chondrocyte spheroid remains available. The purpose of this review was to assess a new emerging modality for cartilage repair and the currently available scientific support for this technique to provide guidance strategy in this evolving field of cartilage repair.

## Figures and Tables

**Table 1:** Critical appraisal of randomized studies.

Lead Author (Year)	Level of Evidence	Random Sequence Generation	Allocation Concealment	Selective Reporting	Other Sources of Bias	Blinding (Participants and Personnel)	Blinding (Outcome Assessment)	Incomplete Outcome Data
**Niemeyer (2016) [10]**, Becher (2017) [11], Niemeyer (2020) [12]	1b	Low	Low	Unclear	Unclear	High	High	Low
**Niemeyer (2019) [13]**, Hoburg (2020) [14]	1b	Low	Low	Unclear	Unclear	High	High	Low

**Table 2:** Critical appraisal of nonrandomized studies.

Lead Author (Year)	Level of Evidence	MINORS Score ^a^
**Fickert (2012) [15]**	2b	11
**Siebold (2014) [16]**	4	9
**Siebold (2016) [17]**	4	8
**Siebold (2018) [18]**	4	12
**Hoburg (2019) [19]**	3b	19 *
**Grevenstein (2020) [20]**	4	8
**Niemeyer (2020) [21]**	2b	22 *
**Sumida (2021) [22]**	3b	8

^a^ Methodological Index for Nonrandomized Studies (MINORS) scores were out of a possible ideal of 16 for noncomparative studies and 24 for comparative studies (*). The levels of evidence are explained in the legend of Table 1.

**Table 3:** Overview of clinical data of patients treated with autologous chondrocyte implantation using spheroids.

Lead Author (Year)	Patients (*n*)	Lesion Size (cm^2^)	Follow-Up (Months)	KOOS						MOCART	IKDC Subjective Score	Lysholm	Other
				Overall	Pain	Sympt	ADL	Sports	QOL				
**Phase II; Niemeyer (2016) [10]**	75	5.0 ± 1.9	12	73.2 ± 17.6	d 16.4 ± 20.2 ^§^	d 12.9 ± 17.3 ^§^	d 12.4 ± 17.9 ^§^	d 17.1 ± 28.9 ^§^	d 22.6 ± 24.4 ^§^	72.4 ± 13.0	68.0 ± 18.3 ^§^		Safety
**Phase II; Becher (2017) [11]**	75	5.0 ± 1.9	36	d 19.9 ± 16.3 ^§^	d 18.2 ± 18.2 ^§^	d 14.8 ± 17.5 ^§^	d 13.6 ± 16.6 ^§^	d 24.3 ± 26.6 ^§^	d 28.9 ± 23.8 ^§^	75.2 ± 13.4 ^§^	73.2 ± 18.8 ^§^		Safety
**Phase II; Niemeyer (2020) [12]**	75	5.0 ± 1.9	48	d 20.1 ± 17.3	d 18.8 ± 18.2	d 14.0 ± 17.5	d 14.2 ± 17.9	d 23.2 ± 28.9	d 30.1 ± 24.1	75.5 ± 13.1	74.6 ± 18.7		IKDC knee examination, safety
**Phase III; Niemeyer (2019) [13]**	52	2.2 ± 0.7	12	78.7 ± 18.6			89.9 ± 16.5 ^$^	71.6 ± 27.5 ^$^		79 ± 14			Macroscopic repair, histology, safety
			24	81.5 ± 17.3			92.1 ± 13.0 ^$^	74.4 ± 24.9 ^$^		76 ± 16	d 24.2 ± 16.9	d 4.9 ± 4.3 ^m^	
**Phase III; Hoburg (2020) [14]**	52	2.2 ± 0.7	36	83.2 ± 14.9			92.8 ± 12.0	79.2 ± 23.2					Safety
**Niemeyer (2020) [21]**	Patella 45 *	5.4 ± 1.6	60	82.6 ± 14.0	88.3 ± 14.4	87.6 ± 13.9	91.4 ± 10.0	76.0 ± 23.0	70.6 ± 21.5				
	Femur 28 *	6.0 ± 1.7		81.9 ± 18.6									
**Siebold (2014) [16]**	10	7.2 ± 3.5	24	74.4 ± 16.9						13.7 ± 1.8 ^m^	63.9 ± 22.1	74.1 ± 18.7	Kujala
**Hoburg (2019) [19]**	Adolescent 29	4.6 ± 2.4	63.3	82.6 ± 11.6	88.5 ± 10.4	83.1 ± 16.1	94.9 ± 7.4	78.6 ± 20.2	67.6 ± 17.2	74.7 ± 12.0	81.1 ± 17.7	21.0 ± 2.4 ^m^	
	Adult 42 *	4.7 ± 1.2	48.4	84.6 ± 11.7	90.9 ± 8.9	91.5 ± 7.0	94.2 ± 7.9	77.7 ± 21.2	69.0 ± 22.3	77.2 ± 11.2	80.5 ± 15.2	22.3 ± 1.9 ^m^	
**Fickert (2012) [15]**	37	4.4 (1.0–12.0)	12							70	64	82.5 (34–100)	Tegner, VAS pain, SF-36, safety
**Siebold (2016) [17]**	41	4.3 ± 3.4	34 ± 19.2		81 ± 12.9	76.8 ± 16.6	85.1 ± 14.9	55.3 ± 27.7	50.6 ± 23.8		63.0 ± 18.8	79.0 ± 18.0	Tegner, macroscopic repair
**Siebold (2018) [18]**	30	6 ± 3.1	34.8 ± 10.2		82.2 ± 16.1	81.7 ± 12.1	86.3 ± 15.6	71.0 ± 16.0	72.3 ± 16.9	60 ± 21 ^m^	84.2 ± 5.6	77.7 ± 14.6	Tegner, EQ-VAS
**Sumida (2021) [22]**	30	4.4 ± 3.7	14.9 ± 16.3										Macroscopic repair
**Grevenstein (2020) [20]**	5		5.5–16										Histology

Mean ± SD or (range) values are provided. d: Delta; meaning change in the outcome score compared to baseline. * Subgroup analyses from the phase II clinical trial. ^m^ modified version of the score was used. sKOOS: Knee injury and osteoarthritis outcome score. Sympt: Symptoms. ADL: Activities of daily living. Sports: Sports and recreation. QOL: Quality of life. MOCART: Magnetic resonance observation of cartilage repair tissue. IKDC: International knee documentation committee. SF: Short form. EQ-VAS: EuroQol Visual Analogue Scale. ^§^: These results were provided in Niemeyer et al., 2020 [12]. ^$^: These results were provided in Hoburg et al., 2020 [14].

**Table 4:** Weighted averages of clinical outcomes of patients treated with autologous chondrocyte implantation using spheroids.

Score	Lead Author (Year)	Value	Patients (*n*)
**Overall KOOS**	Niemeyer (2020) [12]	77.1 ± 18.6	73
	Hoburg (2020) [14]	83.2 ± 14.9	48
	Siebold (2014) [16]	74.4 ± 16.9	10
	Siebold (2016) [17]	69.76	31
	Siebold (2018) [18]	78.7	30
	Hoburg (2019) [19] *	82.6 ± 11.6	29
	Weighted average	78.2	221
**MOCART**	Niemeyer (2020) [12]	75.5 ± 13.1	69
	Fickert (2012) [15]	70	14
	Hoburg (2019) [19] *	74.7 ± 12	29
	Niemeyer 2016 [10] ^§^	76 ± 16	46
	Weighted average	75.0	158
**IKDC**	Niemeyer (2020) [12]	74.6 ± 18.7	73
	Fickert (2012) [15]	64	37
	Siebold (2014) [16]	63.9 ± 22.1	10
	Siebold (2016) [10]	63 ± 18.8	31
	Siebold (2018) [18]	84.2 ± 5.6	30
	Hoburg (2019) [19] *	81.1 ± 17.7	22
	Weighted average	72.5	203
**Lysholm**	Fickert (2012) [15]	82.5	37
	Siebold (2014) [16]	74.1 ± 18.7	10
	Siebold (2016) [17]	79 ± 18	31
	Siebold (2018) [18]	77.7 ± 14.6	30
	Weighted average	79.4	108

* Only the adolescent subgroup is included as the young adults are a subgroup of the phase II clinical trial [10,12]. ^§^ same study as Hoburg (2020) [14]. KOOS: Knee injury and osteoarthritis outcome score. MOCART: Magnetic resonance observation of cartilage repair tissue. IKDC: International knee documentation committee.
